# Mesh Extraction Surgery and Laser Treatment for Pain After Mid-Urethral Sling Surgery: A Case Series

**DOI:** 10.7759/cureus.51431

**Published:** 2024-01-01

**Authors:** Nobuo Okui, Machiko.Aurora Okui

**Affiliations:** 1 Department of Dentistry, Kanagawa Dental University, Kanagawa, JPN; 2 Department of Urology, Dr. Okui’s Urogynecology and Urology Clinic, Kanagawa, JPN

**Keywords:** complications, pain, mesh extraction, stress urinary incontinence, laser therapy, mid-urethral sling

## Abstract

Stress urinary incontinence (SUI) is the leakage of urine due to abdominal pressure. The primary surgical approach involves the insertion of a mid-urethral sling (MUS) with a mesh, which can occasionally lead to post-operative pain. To address complications, MUS removal is often necessary. We hypothesize that a non-ablative erbium:yttrium aluminum garnet (Er:YAG) laser combined with vagina (vaginal erbium laser (VEL)) and urethra (urethra erbium laser (UEL)) treatments could be a post-MUS removal option. A study involving laser treatment started in 2016 for women with recurrent SUI one year after MUS removal who were not affected by pelvic floor muscle exercises and who did not wish to have MUS reinsertion or urethral injection treatment. Five patients (mean age, 54.5 ± 9.35 years) were enrolled, all receiving laser therapy. The visual analog scale (VAS) was used to assess pain as a primary endpoint, and the one-hour pad test was performed for SUI as a secondary endpoint. The mean pain VAS score changed from 8.57 ± 0.69 to 2.29 ± 1.50 (p = 0.00002) after MUS removal. Furthermore, the VAS score was 0 (p = 0.0034) after VEL + UEL. SUI changed from 4.42 ± 2.9 g on the one-hour pad test during MUS insertion to 66.7 ± 39.0 (p = 0.005) after removal. However, after the VEL + UEL treatment, it was 3.71 ± 5.25 g (p = 0.0035). The pathological tissue collected from the five patients at the time of MUS removal surgery had vacuolization in the part where the artificial material was present in the specimen, with foreign-body giant cells proliferated around it. One year after the MUS removal, mucous membrane regeneration was poor, and tissue thickness was thin. One year after the VEL + UEL treatment, the tissue had normalized mucosa, and there was no inflammation. Our study suggests MUS extraction and VEL + UEL as viable options for treating MUS pain in women.

## Introduction

Stress urinary incontinence (SUI) is the leakage of urine triggered by actions, such as coughing or sneezing, affecting about one in three women aged 18 and older, greatly impacting their quality of life (QOL) [1-5]. First-line surgical treatment involves the insertion of a mid‐urethral sling (MUS) [1-5]. There are three types of MUS: tension-free vaginal tape (TVT), transobturator tape (TOT), and a mid-urethral tissue fixation system (TFS). Another treatment modality consists of fascial sling surgery where fascia is inserted instead of a mesh. MUS has a reported high patient satisfaction but a complication rate of 2.7-9.8% over five years [4]. Treatment for complications includes MUS removal (implementation rate: about 3%) and reinsertion surgery (implementation rate: 2.7-7.8%); however, relative to the complication rate, the percentage of individuals that undergo MUS removal is small [4]. Among the complications, pain associated with the mesh can have a significant impact on subsequent QOL [1-6], accounting for 1-17% of MUS extractions [1-6]. A major reason for MUS-related litigation is pain after transvaginal mesh implantation [6].

The main indications for mesh extraction are pain, urethral obstruction, vaginal epithelial erosion, and dyspareunia. However, pain after MUS surgery is difficult to manage with conservative treatments, except for well-defined causes, such as erosion and exposure [4-6]. Conservative treatments include pelvic floor muscle exercises, oral anti-inflammatory analgesics, and local anesthesia with analgesics and anesthetics. Subsequently, when conservative treatments fail, MUS removal is expected to have a certain degree of success; however, there are few studies on mesh extraction [5]. Existing studies suggest that mesh extractions are underestimated [4-6]. This may be primarily due to some physicians or patients believing that mesh removal will cause SUI recurrence or other side effects. Another reason for mesh extraction underestimation may be the insecurity of meshes. Mesh problems are widespread, with the US Food and Drug Administration issuing a warning in 2011 [7].

We hypothesized that if a safe and effective support method existed as an option after mesh extraction, we would be able to actively recommend mesh extraction to women with mesh problems. Our selection consisted of the non-ablative erbium:yttrium-aluminum-garnet (Er:YAG) SMOOTH® laser, accompanied by a laser tailored for the vagina (vaginal erbium laser (VEL)) and the urethra (urethra erbium laser (UEL)). Previous reports showed that a combination of VEL and UEL improved severe SUI [8-14]. VEL itself is very safe, with few side effects observed [12,14]. An illustrative case of severe SUI underwent combined VEL and UEL treatment, demonstrating MRI-documented changes and the uniformization of urethral circumference [8]. The effects of VEL and UEL, using the SMOOTH modalities, involve the use of a 2940 nm SMOOTH Er:YAG laser with high water affinity. VEL and UEL employ a series of non-ablative micropulses to deliver heat penetration at depths of approximately 400-500 um, stimulating tissue regeneration, collagen formation, and blood vessel development. This process occurs without surface damage and has been observed to affect tissues up to 2 mm deep in the mucosa [8-14].

## Case presentation

Case 1

Case 1 concerned a 48-year-old woman (gravida 1 para 1) with a body mass index (BMI) of 22.5 kg/m^2^. She was treated two years after having received transobturator tape (TOT) MUS for SUI. The TOT was inserted through the middle urethra into the left and right obturator spaces.

At the first visit (T0) to our specialty clinic, pain was measured using a visual analog scale (VAS) with a range of 0-10 [9]. On the VAS, 10 was the most painful and 0 was no pain [9]. At T0, there was tenderness along the mesh on the middle urethra and on the left and right vaginal walls of the urethra, with a VAS score of 10 points. In her journal, she repeatedly used the phrase “feels like a knife sticking into me.” The one-hour pad test [9,14] showed a leakage of 10 g. Her International Consultation on Incontinence Questionnaire-Short Form (ICIQ-SF) score [9,14] was seven points. The patient had undergone tumor resection for right breast cancer without chemotherapy, and female hormone replacement therapy was contraindicated.

In this case series, all cases followed the same treatment protocol. The primary endpoint for MUS excision and laser therapy was pain relief, and the secondary endpoint was a cure of SUI. MUS extractions (M1) were performed in 2017-2018. The patients were assessed one year after the last MUS removal surgery (M-T1). For patients with recurrent SUI, a combination of pelvic floor muscle exercises (pelvic floor muscle training (PFMT)) [9] and local estrogen therapy (LET) [13] was prescribed for six months. However, we did not recommend LET for patients in whom it was contraindicated, similar to Case 1. Patients who still experienced persistent SUI were included in a study and underwent VEL + UEL treatment [9]. The VEL + UEL treatment consisted of three sessions with one-month intervals (L1, L2, and L3). Pain and SUI were both reevaluated one year after the third VEL + UEL treatment (L3) (L-T1). MUS removal followed a procedure similar to that of Case 1 for all patients but was customized according to each individual case. VEL + UEL treatments were conducted using the same protocol as in Case 1 for all patients.

The removal of MUS and VEL + UEL treatment necessitated obtaining informed consent from all patients before the commencement of treatment, and we secured handwritten signatures from each patient.

The most significant parameter influencing M1 was the location of pain. These choices were made at the surgeon’s discretion. In addition to the location of the pain, the anatomical relationship between the mesh and the urethra was also considered.

**Figure 1:**
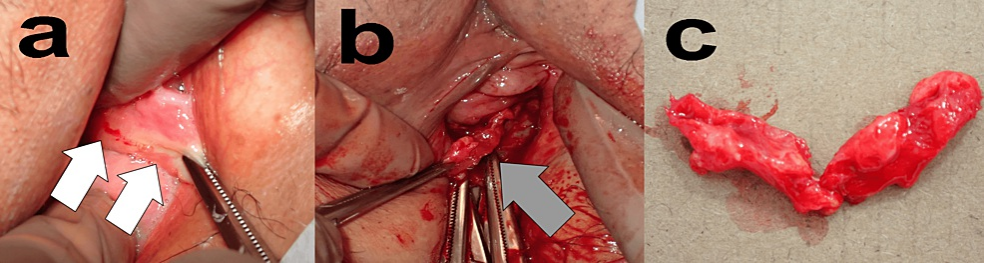
MUS removal surgery procedure (Case 1) a. Assessment of pain location (white arrows) before applying anesthesia. b. After lumbar or general anesthesia, an incision is made into the painful vaginal mucosa and the vaginal wall is peeled to expose the mesh (gray arrow). c. The extracted mesh.

Figure 1 shows an example of the M1 procedure. We checked for tender areas prior to anesthesia for M1. In Figure 1a, the pain was localized, and we observed that the vaginal wall had become hard in that area (white arrows). Subsequently, lumbar anesthesia was performed. In Figure 1b, prior to intervention, a sample was excised from the epithelium to the mesh for histopathological diagnosis. An incision was made in the vaginal wall along the painful part of the mesh and peeled to expose the mesh (gray arrow). We performed these procedures with minimal damage to the surrounding tissue. After removal, the vaginal walls were closed. Figure 1c shows the resected MUS specimen of Case 1.

**Figure 2:**
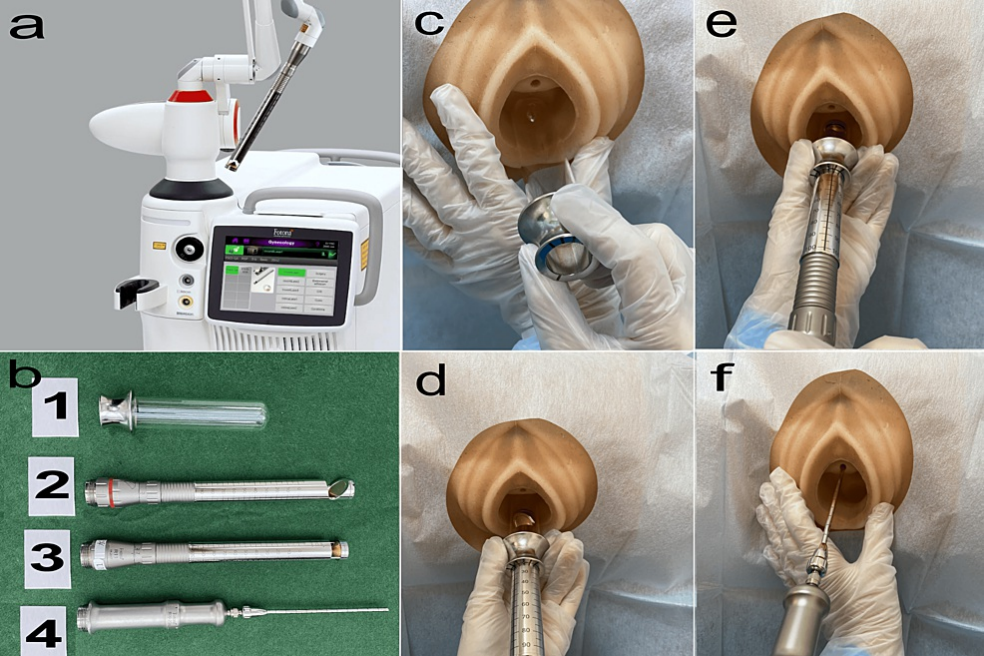
VEL + UEL treatment procedure a. SP Dynamis; Copyright © 2013. Provided courtesy of Fotona d.o.o. (Ljubljana, Slovenia). This image in Figure 2a is provided free of charge by Fotona d.o.o. b. 1, Special glass speculum for laser; 2, PS03 laser probe for the anterior wall of the vagina; 3, R11 laser probe for the entire circumference of the vagina; 4, R09-2Gu laser probe for the entire circumference of the urethra. c. VEL step (glass speculum insertion). d. VEL step (laser irradiation of anterior vaginal wall by PS03). e. VEL step (whole vaginal laser irradiation by R11). f. UEL step (whole urethral laser irradiation with R09-2Gu).

For the VEL + UEL treatment, VEL was performed first, followed immediately by UEL. For VEL, the vagina, labia, and urethra were disinfected with iodine. An 8% xylocaine spray (Sandoz KK, Tokyo, Japan) was applied for 15 minutes for anesthesia. Laser irradiation was performed with Renovalase (SP Dynamis Fotona d.o.o, Ljubljana, Slovenia, Figure 2a), starting with VEL and proceeding to UEL. The prepared devices included a special glass vaginal speculum dedicated to the laser probes PS03, R11, and R09-2 Gu, as well as handpieces (Figure 2b for PS03, R11, and R09-2 Gu). Each handpiece was connected to the SP Dynamis laser. In the VEL step, the glass speculum was inserted into the vagina (Figure 2c), and the anterior vaginal wall was scanned with a PS03 laser probe with a spot size of 7 mm, pulse fluence of 6 J/cm^2^, and frequency of 2.0 Hz (Figure 2d). The area was then irradiated every 5 mm. This procedure was repeated three times. Subsequently, the R11 laser probe was used to apply laser treatment at 5 mm intervals along the entire 360-degree vaginal canal. This treatment utilized a spot size of 7 mm, a pulse fluence of 3.00 J/cm^2^, and a frequency of 2.0 Hz (Figure 2e). This procedure was repeated twice. For UEL, an R09-2 Gu laser probe designed for the urethra was used via a catheter after the withdrawal of residual urine from the bladder. The laser treatment settings were R09-2 Gu, SMOOTH, 1.4 Hz, 1.5 J/cm^2^, and four stacks from the urethral meatus to the proximal end in 2.5 mm increments (Figure 2f). This treatment was repeated four times. The entire process of VEL + UEL was completed in approximately 30 minutes. Three sessions of the VEL + UEL treatment were performed in intervals of one month (L1, L2, and L3), and both pain and SUI were reassessed one year after the third VEL + UEL treatment (L3) (L-T1).

**Figure 3:**
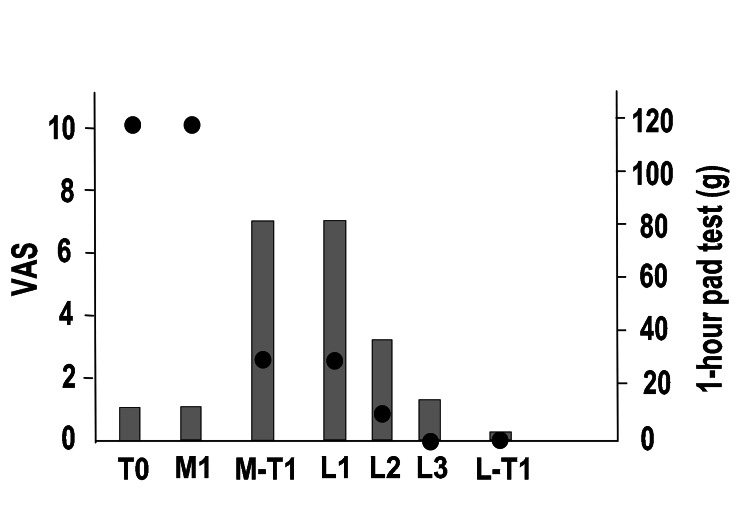
Overall course of pain and SUI in Case 1 Left vertical axis: degree of pain (VAS), right vertical axis: one-hour pad test, horizontal axis: time, black circles: VAS, gray bars: one-hour pad test SUI: stress urinary incontinence, VAS: visual analog scale pain score (0: no pain to 10: greatest pain), T0: first visit, M1: first mid-urethral sling (MUS) removal surgery, M-T1: 1 year after MUS removal surgery, L1: first laser treatment, L2: second laser treatment, L3: third laser treatment, L-T1: one year after the third laser treatment (L3).

Figure 3 shows the entire course of the procedure. For MUS removal, we made a median incision on the anterior vaginal wall under lumbar anesthesia and completely removed the mesh and anchor (M1). The patients underwent evaluation one year after the last MUS removal surgery (M-T1).

We prescribed PFMTs [9] for a duration of six months to prevent recurrent SUI. In the case of persistent SUI in Case 1, we prospectively enrolled and initiated treatment with VEL + UEL.

One year after the operation in Case 1, the VAS improved to three points, but the one-hour pad test deteriorated to 80 g, and the ICIQ-SF worsened to 16 points (M-T1). PFMTs were performed for six months, but the SUI did not improve. The ICIQ-SF did not indicate MUS reinsertion, and hence, UEL + VEL was performed three times (L1, L2, and L3). During this time, a significant decrease in the VAS score and improvement in SUI were observed. One year after L3 (L-T1), the VAS became 0, the 1-hour pad test was 1 g, and the ICIQ-SF score was five points.

**Figure 4:**
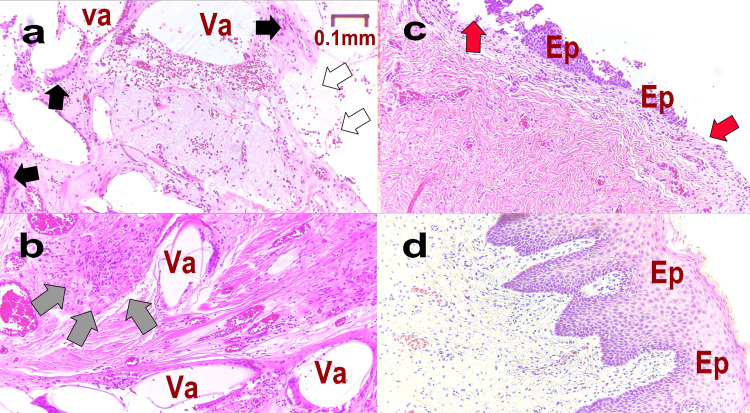
Pathological tissue of Case 1 Pathology is assessed at each stage as follows: M1: at the time of mid-urethral sling (MUS) removal surgery (a. epithelium, b. deep); M-T1: one year after MUS removal (c); L-T1: one year after the third laser treatment (d). Va: vacuole formed when the mesh fell off during pathological specimen preparation, Ep: mucosa, white arrow: shedding of mucosal epithelium, gray arrow: lack of mucosal epithelium

Figure 4 shows the pathology at M1 (Figure 4a, b), M-T1 (Figure 4c), and L-T1 (Figure 4d). In Figure 4a, the area containing the artificial mesh became detached during the preparation of the specimen, resulting in a blank formation (Va). Multinucleated giant cells (black arrows) were observed around many empty cannons (Va), and the epithelium was sloughed off (white arrow). Figure 4b confirms vacuolation (Va) and foreign body granulation (gray arrow). Consistent with pain caused by MUS, recurrent inflammation indicated suppression of normal cell proliferation and proliferation of poor granulation tissue. In Figure 4c, although the empty cells have disappeared, there is a partial mucosal defect (red arrow) and slight regeneration of the mucosal epithelium (Ep). In Figure 4d, normal mucosa (Ep) proliferates, and multinucleate giant cells and empty cells are absent. This shows that the patient’s condition has normalized.

Case 2

Case 2 concerned a 72-year-old woman (gravida 2 para 2) with a BMI of 26.0 kg/m^2^ who had undergone TVT MUS for SUI at the age of 60 years. Twelve years had passed since she first came to our facility, during which time the vaginal wall pain on the right side of the mid-urethra had persisted. In her journal, she wrote “When I touch tissue paper after urinating, I feel the pain of being pricked with needles” and “When I sit in a chair, I feel the pain of several needles.”

**Figure 5:**
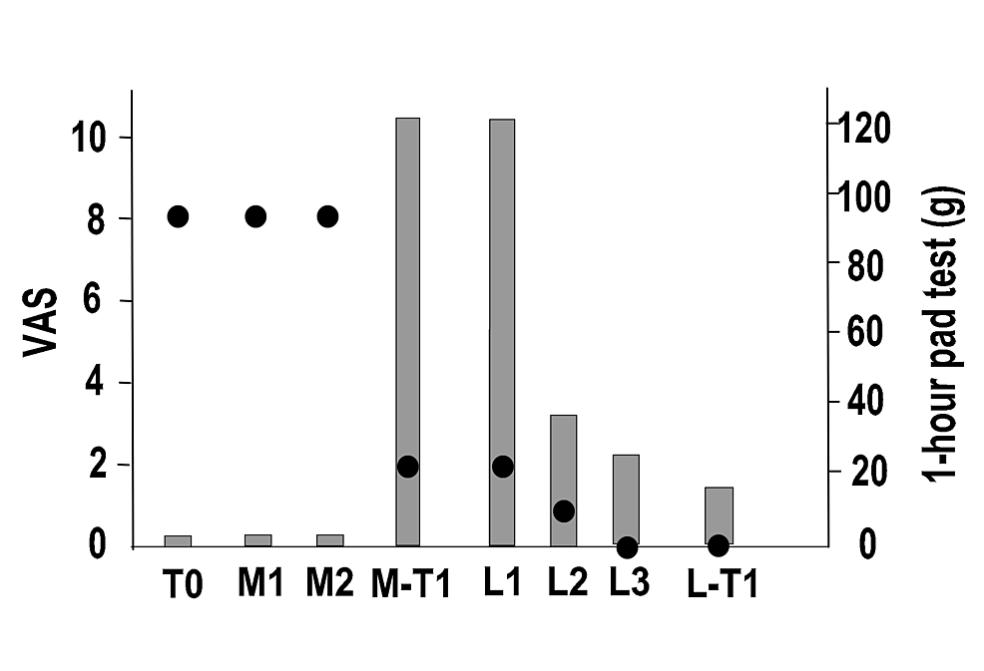
Overall course of pain and SUI in Case 2 Left vertical axis: degree of pain (VAS), right vertical axis: one-hour pad test, horizontal axis: time, black circles: VAS, gray bars: one-hour pad test SUI: stress urinary incontinence, VAS: pain score on a visual analog scale (0: no pain to 10: maximal pain), T0: first visit, M1: first surgery to remove the mid-urethral sling (MUS), M2: second surgery to remove the MUS, M-T1: one year after M2, L1: first laser treatment, L2: second laser treatment, L3: third laser treatment, L-T1: one year after the third laser treatment (L3)

Figure 5 shows the entire process of the procedure. At T0, the VAS pain score was eight points, but the one-hour pad test was 1 g and no SUI was observed. Moreover, the ICIQ-SF score was 0 and the SUI had no effect on the QOL. The patient had already received PFMT and LET guidance at another hospital, but it was not effective. For MUS removal, a median incision was made on the anterior wall of the vagina, and the right mesh was removed from the periurethral area in the first operation (M1); however, the VAS did not improve, and the left mesh was removed in the second operation (M2). Similar to the procedure followed for Case 1, we identified the painful area before anesthesia and marked it on the vagina. The first procedure resulted in a bleeding volume of 20 ml. During the second procedure, the amount of bleeding was 220 ml. Immediately after M2, the VAS score improved to two points, but the patient developed SUI. At M-T1, the VAS score was two points, and the one-hour pad test deteriorated to 120 g. The ICIQ-SF score was 16 points. The patient had a strong aversion to foreign objects and did not wish to have MUS reinsertion or urethral injection treatment. Hence, UEL + VEL was performed three times (L1, L2, and L3). Improvements began to appear from L2, and at L3, the VAS score improved to 0 points, and the one-hour pad test improved to 5 g. At L-T1, the VAS score was 0, the one-hour pad test was 15 g, and the ICIQ-SF score was 12 points. We are considering the possibility of MUS reinsertion in the future.

**Figure 6:**
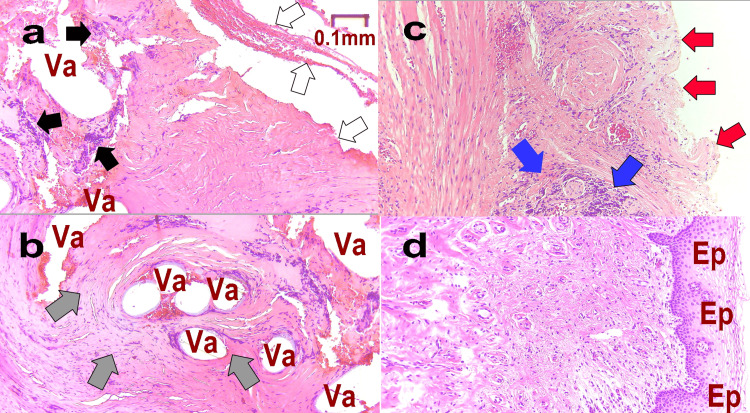
Pathological tissue of Case 2 Pathology is assessed at each stage as follows: M1: at the time of mid-urethral sling (MUS) removal surgery (a. epithelium, b. deep); M-T1: one year after MUS removal (c); L-T1: one year after the third laser treatment (d). Va: vacuole formed when the mesh fell off during the pathological specimen preparation, Ep: mucosa, black arrows: foreign-body giant cells, white arrows: most of the epithelium has fallen off, gray arrows: foreign body defective granulation, red arrows: missing mucous membrane, blue arrows: defective granulation

Figure 6 shows the histopathology of Case 2. Figure 6a shows a sample containing epithelium at M1. There are many blank formations (Va) after the presence of the mesh. Foreign-body giant cells are observed around it (black arrows). Most of the epithelium has become detached (white arrows). Figure 6b shows a deeper area, with a large foreign body defective granulation at the edge of multiple vacuolations (gray arrows). Direct palpation of the pathology sample revealed very stiff tissue, consistent with a lack of elasticity. These findings indicate a high degree of foreign body reaction. Figure 6c shows M-T1, in which tissue is regenerated but the mucous membrane is missing (red arrows), and defective granulation tissue is observed (blue arrows). Figure 6d shows L-T1, with a normal mucosal proliferation (Ep) and absence of multinucleated giant cells and empty cells.

Case 3

Case 3 concerned a 56-year-old woman (gravida 2 para 2) with a BMI of 28.2 kg/m^2^ who had undergone hysterectomy at the age of 44 years for uterine prolapse. Subsequently, she developed SUI. At 54 years of age, she underwent TFS MUS insertion. Immediately afterward, pain continued along the mesh on the right side of the vagina. In her journal, she wrote “a pain that I cannot forget no matter what I do,” “the reason why I quit my job,” and “the pain reverberated from the groin to the entire pelvis when carrying luggage at work.”

**Figure 7:**
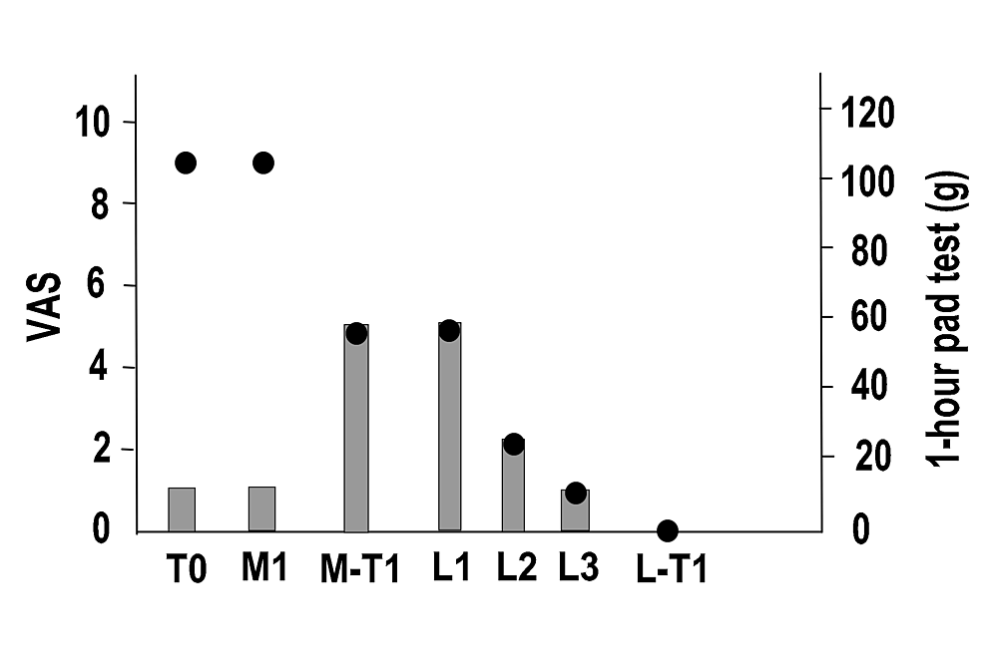
Overall course of pain and SUI in Case 3 Left vertical axis: degree of pain (VAS), right vertical axis: one-hour pad test, horizontal axis: time, black circles: VAS, gray bars: one-hour pad test SUI: stress urinary incontinence, VAS: pain score on a visual analog scale (0: no pain to 10: maximal pain), T0: first visit, M1: first mid-urethral sling (MUS) removal surgery, M-T1: one year after MUS removal surgery, L1: first laser treatment, L2: second laser treatment, L3: third laser treatment, L-T1: one year after the third laser treatment (L3)

Figure 7 shows the entire process. At T0, her VAS pain score was nine points, the one-hour pad test was 5 g, and ICIQ-SF was four points. M1 was performed by creating a midline anterior vaginal incision and removing the entire mesh. As performed for Case 1, lumbar anesthesia was administered after confirming the pain. Care was taken during MUS removal to avoid damaging the surrounding tissue. There was minimal bleeding during the procedure. Immediately after M1, her VAS score was five points, the one-hour pad test was 5 g, and the ICIQ-SF score was eight points. At M-T1, her one-hour pad test was 60 g, and the ICIQ-SF score was 17 points. LET and PFMT were continued for six months with no improvement. Moreover, because of the pain, the patient refused MUS reinsertion. Therefore, UEL + VEL was performed three times (L1, L2, and L3). Improvements were seen from the first treatment, with a VAS score of 0 points at L-T1, a one-hour pad test of 0 g, and an ICIQ-SF score of three points.

**Figure 8:**
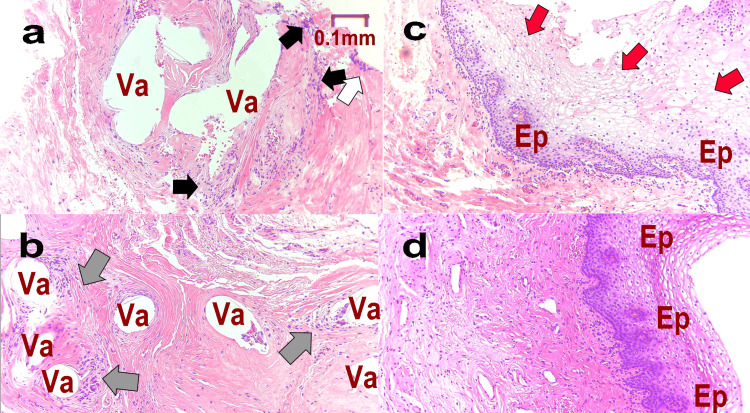
Pathological histology of Case 3 Pathology is measured at each stage as follows: M1: at the time of mid-urethral sling (MUS) removal surgery (a. epithelium, b. deep); M-T1: one year after MUS removal (c); L-T1: one year after the third laser treatment (d). Va: vacuole formed when the mesh fell off during pathological specimen preparation, Ep: mucosa, black arrows: foreign-body giant cells, white arrows: shedding of mucous membrane, gray arrows: foreign body granuloma, red arrows: edema

Figure 8 shows the histopathology of Case 3. Figures 8a and 8b show the pathology at M1. In both pathologic specimens, numerous void formations indicate the presence of a mesh (Va). Multinucleated giant cells are observed around it (black arrows). A large number of foreign body granulomas have formed around the mesh (gray arrows). The mucous membrane has become detached (white arrows). At M-T1, the tissue has regenerated, and the mucous membrane is observed, but there is overall edema, which is abnormal (red arrows, Figure 8c). At L-T1, normal mucosa is seen to have proliferated and regenerated (Figure 8d).

Case 4

Case 4 concerned a 47-year-old woman (gravida 2 para 2) with a BMI of 27.0 kg/m^2^. She had stage 2 endometriosis with a contraindication for LET. She had undergone TOT MUS insertion for SUI at the age of 46 years. Subsequently, she felt pain in the wound of the right obturator membrane and in the area of the vagina, where the right mesh was inserted. An ulcer was observed in the vagina due to the mesh. In her journal, she recorded “pain when urinating,” “pain when touching tissue paper after urinating,” and “pain with several needles in her body.”

**Figure 9:**
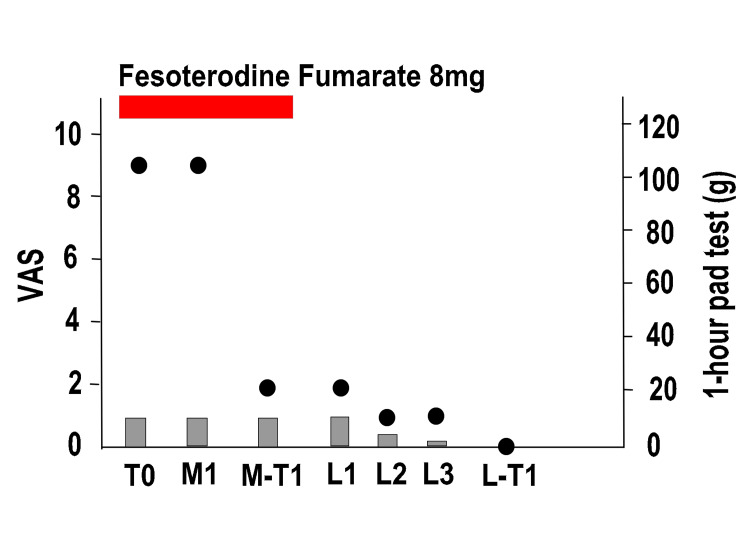
Overall course of pain, SUI, and OAB in Case 4 Left vertical axis: pain level (VAS), right vertical axis: one-hour pad test, horizontal axis: time, black circles: VAS, gray bars: one-hour pad test SUI: stress urinary incontinence, VAS: pain score on a visual analog scale (0: no pain to 10: maximal pain), T0: first visit, M1: first mid-urethral sling (MUS) removal surgery, M-T1: one year after MUS removal surgery, L1: first laser treatment, L2: second laser treatment, L3: third laser treatment, L-T1: one year after the third laser treatment (L3)

The patient had a strong urge to urinate and was using fesoterodine fumarate 4 mg daily. The patient was not using mirabegron owing to hypertension. PFMT had no effect. At T0, one year after TOT insertion, her VAS pain score was nine points, 1-hour pad test was 7 g (mild SUI), overactive bladder symptom score (OABSS) [14] was 10 points (moderate overactive bladder: OAB), and ICIQ-SF score was 12 points. Figure 9 shows the entire process of the procedure. M1 was performed through a midline anterior vaginal wall incision. A skin incision was then made from the insertion site to the mesh in the vagina along the right plain from just below the urethra and to the right obturator membrane, and the right half of the mesh was removed. The procedure was performed as described for Case 1. Care was taken to avoid tissue damage during MUS removal; however, there was bleeding of 180 ml. At M-T1, her VAS score improved to two points, one-hour pad test was 7 g, OABSS was two points, and ICIQ-SF was five points. There was no change in SUI. The patient refused additional MUS reinsertion. After stopping the use of fesoterodine fumarate 4 mg for three months, UEL + VEL was performed three times (L1, L2, and L3). Because of the discontinuation of fesoterodine fumarate, the OABSS returned to five points at L1, but the laser treatment led to significant improvement from the first session. The patient’s OABSS and ICIQ-SF score became 0 points.

**Figure 10:**
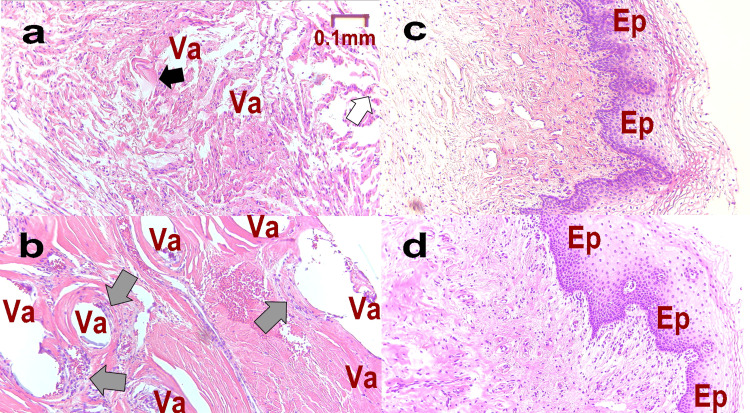
Pathological tissue of Case 4 Pathology is assessed at each stage as follows: M1: at the time of mid-urethral sling (MUS) removal surgery (a. epithelium, b. deep); M-T1: one year after MUS removal (c), L-T1: one year after the third laser treatment (d) Va: vacuole formed when the mesh fell off during pathological specimen preparation, Ep: mucosa, black arrow: ossification, white arrow: shedding of mucous membrane, gray arrows: foreign body granuloma

In the histopathological photographs shown in Figure 10, in M1, the epithelium has become detached, and numerous vacuoles (Va) containing a mesh and foreign-body giant cells (gray arrows) are observed (Figures 10a, b). These are partially accompanied by ossification (black arrow). In M-T1, the epithelium has regenerated, and the foreign-body giant cells have disappeared (Figure 10c). In L-T1, regeneration of the epithelium is showing clear progress, and mild hyperplasia of collagen fibers is observed under the epithelium. No inflammation within the tissue is observed (Figure 10d).

Case 5

Case 5 concerned a 55-year-old woman (gravida 2 para 2) with a BMI of 24.8 kg/m^2^. She had undergone TFS surgery for SUI at the age of 51 years. After surgery, she experienced pain in the vaginal urethra on the left and right sides and in the midline. Sitting in a chair aggravated the pain. In her journal, she wrote that when she put her weight on it, such as sitting on a chair, she felt a knife-stabbing pain.

**Figure 11:**
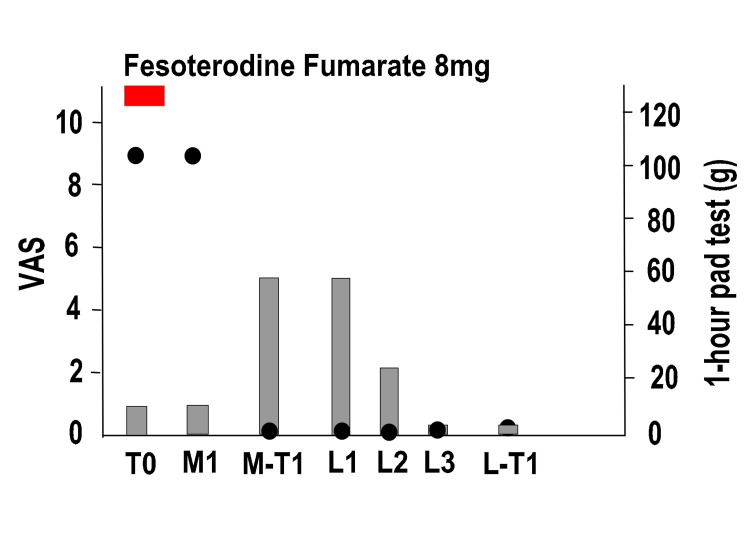
Overall course of pain, SUI, and OAB in Case 5 Left vertical axis: degree of pain (VAS), right vertical axis: one-hour pad test, horizontal axis: time, black circles: VAS, gray bars: one-hour pad test, red rectangle: duration of fesoterodine fumarate administration SUI: stress urinary incontinence, VAS: pain score on a visual analog scale (0: no pain to 10: maximal pain), T0: first visit, M1: first mid-urethral sling (MUS) removal surgery, M-T1: one year after MUS removal surgery, L1: first laser treatment, L2: second laser treatment, L3: third laser treatment, L-T1: one year after the third laser treatment (L3)

These pain characteristics correlated with the TFS mesh. Figure 11 shows the entire process. At T0, the VAS was nine points, one-hour pad test was 5 g (mild SUI), OABSS was eight points (moderately OAB), and ICIQ-SF score was eight points. LET had been implemented but had not shown any improvements. Furthermore, 8 mg daily of fesoterodine fumarate was administered orally for three months, with no improvement. A mesh total extirpation was performed (M1). In addition, similar to Case 1, we confirmed the pain and proceeded with lumbar anesthesia. During MUS removal, we took precautions to avoid tissue trauma. However, there was strong adhesion around the TFS anchors. The bleeding during the procedure amounted to 30 ml. The pain and OAB symptoms disappeared immediately after the operation. However, the SUI worsened to 60 g on the one-hour pad test and 12 points on the ICIQ-SF. MUS reinsertion was not desired for exacerbated SUI; therefore, UEL + VEL was performed three times (L1, L2, and L3). The patient improved significantly from L1, with a VAS score of 0 points, one-hour pad test of 2 g, OABSS of 0 points, and an ICIQ-SF score of four points. At L-T1, the pain and SUI remained improved.

**Figure 12:**
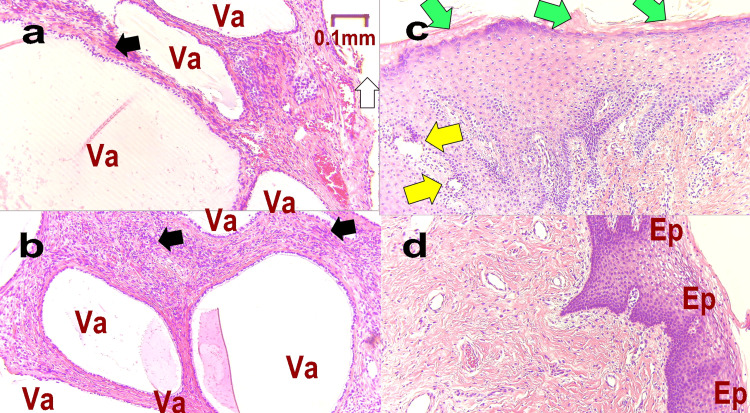
Histopathological tissue in Case 5 Pathology is assessed at each stage as follows: M1: at the time of mid-urethral sling (MUS) removal surgery (a. epithelium, b. deep); M-T1: one year after MUS removal (c); L-T1: one year after the third laser treatment (d) Va: vacuole formed when the mesh fell off during pathological specimen preparation, Ep: mucosa, black arrows: foreign-body giant cells, white arrow: shedding of mucous membrane, green arrow: fibrin (scarring), yellow arrow: cyst formation

Figure 12 shows the pathology of Case 5. Figures 12a and 12b show M1, in which a large vacuolization (Va) is observed in the area where the mesh was inserted, and a large number of foreign-body giant cells (black arrows) are observed. The epithelium is sloughed off (white arrow). Pathologically, at M-T1 in Figure 12c, the vacuoles have disappeared, but macroscopically, scars are observed to exist uniformly (green arrows). Furthermore, there is heterogeneous benign cyst formation (yellow arrows). Figure 12d shows L-T1 where the mucosa appears thickened and normalized.

Table 1 shows the patient characteristics. The mean age was 56.3 years (47-72 years) and the duration of indwelling MUS was 4.2 years (1-12 years). One patient had a history of breast cancer and one had uterine prolapse. Regarding comorbidities, one patient had hypertension and one had diabetes.

**Table 1:** Patient characteristics MUS: mid-urethral sling, BMI: body mass index, SD: standard deviation

	Values
Age (years), mean ± SD	56.3 ± 8.54
First surgery to remove MUS after insertion of MUS (years), mean ± SD	4.2 ± 4.01
BMI (kg/m^2^), mean ± SD	25.44 ± 2.13
Medical history	
Breast cancer	1 case
Uterine prolapse	1 case
Comorbidities	
Hypertension	1 case
Diabetes	1 case
Smoking	1 case

Table 2 shows the changes in VAS and one-hour pad test after MUS resection in the five cases. The average VAS score was 9.0 ± 0.63 before MUS removal, but it improved to 2.4 ± 1.64 1 year after the removal. However, there were cases with recurrence of SUI, and the average one-hour pad test deteriorated from 5.6 ± 2.93 g before MUS removal to 65.6 ± 35.6 g one year after removal. One year after L3, the VAS and SUI improved to 0 and 3.6 ± 5.74 g, respectively.

**Table 2:** VAS, one-hour pad test, OABSS, and ICIQ-SF VAS: pain score on a visual analog scale (0: no pain to 10: maximal pain), OABSS: Overactive Bladder Symptom Score, ICIQ-SF: International Consultation on Incontinence Questionnaire-Short Form, M1: first mid-urethral sling (MUS) removal surgery, M-T1: one year after MUS removal surgery, L-T1: one year after the third laser treatment (L3), SD: standard deviation

Item	Case	M1	M-T1	L-T1	Significant difference (M1 and M-T1)	Significant difference (M-T1 and L-T1)
VAS, mean ± SD	All cases	9.0 ± 0.63	2.4 ± 1.64	0	p = 0.0006	p = 0.012
1-hour pad test, mean ± SD	All cases	5.6 ± 2.93 g	65.6 ± 35.6 g	3.6 ± 5.74	p = 0.017	p = 0.009
OABSS, mean ± SD	Case 4 and Case 5	9.0 ± 1.0	1.0 ± 1.0	0	-	-
ICIQ-SF, mean ± SD	All cases	6.2 ± 4.01	12.6 ± 4.08	3.2 ± 1.72	p = 0.086	p = 0.0009

In the two cases in which OAB appeared during the observation period, MUS excision surgery improved OABSS from 9.0 to 1.0 and laser treatment improved it to 0. Owing to the small number of cases, statistical analysis was not possible.

Only Case 4 showed improvement in the ICIQ-SF score after MUS removal because the cause of OAB/UUI induced by MUS insertion or pain disappeared after the mesh was removed. In the other cases, the recurrence of SUI worsened the ICIQ-SF score. There was deterioration from M1 to M-T1, but it was not statistically significant. The difference in the ICIQ-SF score from M1 to L-T1 was significant; therefore, laser therapy significantly improved the ICIQ-SF score.

## Discussion

There is no consensus on how to treat pain after MUS surgery. In our study, we were able to present evidence that the combination of MUS removal and laser therapy shows favorable results. We believe that this protocol may provide treatment options for complications after MUS surgery.

First, we investigated complications and MUS removal rates after surgery. Keltie et al. [1] found a 9.8% complication rate in an eight-year study of 92,246 MUS insertion patients. Unger et al. [2] reported 89 sling revision surgeries out of 3,307 cases with urinary issues. Gurol-Urganci et al. [3] found removal rates of 1.4% at one year, 2.7% at five years, and 3.3% at nine years for 95,000 UK women. Low MUS removal surgeries relative to complications might be due to underestimation [4].

In our study, we observed that Case 2 hesitated to undergo MUS removal for a duration of 12 years. This patient believed that if SUI recurred after MUS removal, she would have to undergo another procedure to insert a synthetic device. Her mistrust of the implanted device significantly delayed her decision-making process. We believe that this psychological issue serves as evidence supporting the underestimation of the necessity of MUS removal.

Second, we compared the effects of MUS extraction surgery with previous studies. Agnew et al. [5] treated 47 post-MUS insertion patients, including eight who received pain relief post-removal. Hou et al. [15] performed suburethral mesh removal in 54 patients, reducing VAS scores from 5.3 to 1.5, with 67% reaching a VAS score of 0. Mengerink et al. [4] reported a study of 31 patients, where pain decreased from 7.8 ± 1.9 to 4.5 ± 3.2 during a 12-month follow-up, with 23% achieving a VAS score of 0. Twenty-two patients had pain scores of 7.8 ± 1.9 before MUS extraction and 4.5 ± 3.2 after 12 months. Zoorob et al. [6] also mentioned 23% with a VAS score of 0.

In our study, VAS scores improved from 9.0 ± 0.63 at M1 to 2.4 ± 1.64 at M-T1, with only one patient reaching a VAS score of 0. However, the effectiveness of MUS excision surgery varies. Several factors, including scar tissue, foreign body reaction, inflammation, infection, and surgical skill, contribute to pain. Histopathological tissue analysis revealed the mesh's presence during MUS resection, forming vacuoles and attracting foreign-body giant cells. Tissue biopsied one year post-mesh removal showed varying regenerative patterns, indicating insufficient tissue regeneration.

Third, we assessed the downsides of mesh removal, including SUI recurrence and OAB/UUI. For MUS removal type, 50% with partial urethral mesh removal experienced increased SUI, and 61% with complete removal reported worsened SUI, showing no significant difference [4]. Ramart et al. [16] examined 117 patients post-MUS extraction, with 38.6% (TVT) and 34.0% (TOT) experiencing severe SUI one year after removal, requiring additional treatments, such as TVT and TOT MUS. While these studies vary, they stress the need for post-MUS excision SUI treatment. Regarding OAB/UUI, Pikaart et al. [17] reported that an elderly woman in her late 60s experienced 100 ml of bleeding during surgery and presented with urinary frequency and urgency postoperatively.

In our study, two patients with pain after MUS insertion had OAB after MUS removal that did not improve even after using OAB drugs, such as vibegron and fesoterodine fumarate. It should also be noted that surgical excision is difficult. In Case 2, the patient was in her 70s and required two surgeries with a large amount of normal tissue excised owing to the large amount of bleeding, especially in the second MUS removal operation. We thought that Case 2 is similar to Pikaart et al.'s case.

Fourth, we evaluated the effectiveness of mesh and laser treatments. Chapple et al. [18] found that the time between MUS insertion and removal surgery shortened significantly, possibly due to healthcare providers’ increased awareness of complications. It is unclear whether patients and surgeons prefer mesh reinsertion after SUI recurrence post-MUS excision. Okui et al. [14] reported that VEL is effective for patients who decline mesh kits, with a study comparing TVT and VEL showing concerns about mesh kits among non-TVT opters. Erel et al. [10] assessed the effects of VEL on patients who had undergone failed TOT/TVT surgery. They observed improved ICIQ-SF scores in both groups, indicating that VEL could serve as an alternative for reoperation in cases of failed MUS procedures. UEL, intraurethral SMOOTH Er:YAG laser, helped 22 type III SUI women with significant ICIQ-SF scores and 1-hour pad test improvements [19].

In our study, we used a combination of VEL and UEL. The laser treatment reduced pain (VAS score from 2.4 ± 1.64 to 0), improved the one-hour pad test (from 65.6 ± 35.6 g to 3.6 ± 5.74 g), and decreased OAB symptoms (OABSS reduced to 0 from 1.0 ± 1.0) for two patients. In addition, the ICIQ-SF, a QOL indicator, significantly improved from 12.6 ± 4.08 to 3.2 ± 1.72. These results suggest the potential benefits of laser therapy post-MUS removal. We analyzed pathological findings in a previously unexplored area, comparing MUS resection specimens at one year (M-T1) and L-T1. The aberrant tissue patterns observed after mesh removal indicated that laser therapy contributed to tissue improvement, aligning with the results anticipated from Okui et al.'s [14] and Gaspar et al.'s [20] vaginal wall pathology study. Both VEL and UEL were well tolerated in our study, with no reported complaints from the patients, such as burning during urination or dysuria. Furthermore, there were no observed indications of a risk of urethral stricture associated with laser therapy, which is consistent with previous research [8-14,19,20].

## Conclusions

Five cases of SUI who underwent MUS insertion surgery and subsequently suffered from pain were studied. Pain improved in all cases after MUS extraction surgery. In addition, in some women with OAB that appeared after MUS insertion, OAB improved. After MUS resection, there was one woman with recurrence of SUI and exacerbation of OAB, which improved with the VEL + UEL treatment. Four of the five patients were satisfied, and one considered additional treatment. In the pathological specimen, the MUS-excised specimen had vacuolization at the site of the mesh, and foreign-body giant cells were observed around it, indicating a foreign body reaction. Even after one year of excision, the cells in the excised area were weak and abnormal. The VEL + UEL treatment normalized the tissue and thickened the mucosal layer. Given these facts, if laser treatment is available as an option, we recommend proactive MUS extraction for patients experiencing pain after MUS insertion. The indications for surgical treatment of female SUI using synthetic mesh should be more precisely respected and responsively used.
